# Tracing the color: quantitative trait loci analysis reveals new insights into red-flesh pigmentation in apple (*Malus domestica*)

**DOI:** 10.1093/hr/uhae171

**Published:** 2024-06-27

**Authors:** Pierre Bouillon, Anne-Laure Fanciullino, Etienne Belin, Sylvain Hanteville, Hélène Muranty, Frédéric Bernard, Jean-Marc Celton

**Affiliations:** Univ Angers, Institut Agro, INRAE, IRHS, SFR QUASAV, F-49000 Angers, France; IFO, 49140, Seiches sur le Loir, France; Univ Angers, Institut Agro, INRAE, IRHS, SFR QUASAV, F-49000 Angers, France; Univ Angers, Institut Agro, INRAE, IRHS, SFR QUASAV, F-49000 Angers, France; Univ Angers, Institut Agro, INRAE, IRHS, SFR QUASAV, F-49000 Angers, France; Univ Angers, Institut Agro, INRAE, IRHS, SFR QUASAV, F-49000 Angers, France; IFO, 49140, Seiches sur le Loir, France; Univ Angers, Institut Agro, INRAE, IRHS, SFR QUASAV, F-49000 Angers, France

## Abstract

Red-flesh color development in apple fruit is known to depend upon a particular allele of the MdMYB10 gene. While the anthocyanin metabolic pathway is well characterized, current genetic models do not explain the observed variations in red-flesh pigmentation intensity. Previous studies focused on total anthocyanin content as a phenotypic trait to characterize overall flesh color. While this approach led to a global understanding of the genetic mechanisms involved in color expression, it is essential to adopt a more quantitative approach, by analyzing the variations of other phenolic compound classes, in order to better understand the molecular mechanisms involved in the subtle flesh color variation and distribution. In this study, we performed pedigree-based quantitative trait loci (QTL) mapping, using the FlexQTL™ software, to decipher the genetic determinism of red-flesh color in five F1 inter-connected families segregating for the red-flesh trait. A total of 452 genotypes were evaluated for flesh color and phenolic profiles during 3 years (2021–2023). We identified a total of 24 QTLs for flesh color intensity and phenolic compound profiles. Six QTLs were detected for red-flesh color on LG1, LG2, LG8, LG9, LG11, and LG16. Several genes identified in QTL confidence intervals were related to anthocyanin metabolism. Further analyses allowed us to propose a model in which the competition between anthocyanins and flavan-3-ols (monomer and oligomer) end-products is decisive for red-flesh color development. In this model, alleles favorable to high red-flesh color intensity can be inherited from both white-flesh and red-flesh parents.

## Introduction

Most modern apple cultivars originate from a limited number of elite genotypes [1]. These cultivars can be broadly categorized into red and yellow skin-colored fruits. Once this distinction is established, achieving innovative breakthroughs in fruit phenotype becomes challenging. Red-flesh apples, characterized by their appealing color, have garnered significant attention from breeders. The breeding of red-flesh apples dates back to 1897 and this phenotype finds its origin in the wild species *Malus sieversii f. niedzwetzkyana* [2].

Fruit flesh color is closely associated with anthocyanin accumulation [3], a water-soluble phenolic compound that imparts red, purple, and bluish hues to many flowers and fruits. The consumption of anthocyanins is known to have beneficial effects on human health, which underlines the importance of improving their content in plant products [4].

Anthocyanins belong to the antioxidant compound class that also contains flavan-3-ols (soluble and condensed tannins), flavonols, hydroxycinnamic acids, and dihydrochalcones, which serve as the primary polyphenols [5]. Previous research has shown that domestication and selection processes have influenced the biochemical profile of white-flesh apple fruits [6], including tannins (flavan-3-ols), organic acids, and phenolic acids, while some phenolics remained unaffected by selection [7]. Recent studies on red-flesh apples found that these fruits generally contain higher amounts of anthocyanins, dihydrochalcones, phenolic acids, and organic acids, but lower amounts of flavan-3-ols compared to white-flesh fruits [8]. Alternatively, some cultivars exhibit higher levels of both anthocyanins and flavan-3-ols [9]. These contrasted results highlight the need to consider the fruit phenolic profile in the breeding process.

The sequential steps leading to the biosynthesis of anthocyanins are well conserved among plant species [10] and involve structural genes and transcription factors (TFs) regulating anthocyanin accumulation [11]. Theses TFs comprise the myelobla-stosis family (MYB), the basic helix–loop–helix (bHLH) and the tryptophan-aspartic acid repeat (WDR) proteins. Generally, members of these three families of regulatory factors are self-associated through a MYB-bHLH-WD40 (MBW) complex to activate flavonoid late biosynthesis genes [12]. Additionally, WRKY class TFs have been linked to anthocyanin synthesis by regulating vacuolar acidification [12]. Until now, two different types of red-flesh apples have been characterized. In cultivars displaying the type 1 red-flesh phenotype, anthocyanins are distributed throughout the fruit, from fruit set through maturity. This phenotype is associated with red pigmentation of leaves, stems, roots, and flowers. The type 2 red-flesh apple phenotype is characterized by green vegetative tissue, yellow-orange fruit skin, and a red pigmentation that occurs only in the fruit flesh during later stages of fruit development [13]. Type 1 red-flesh is dependent upon the presence of a particular allele of the MdMYB10 gene (historically termed ‘R’ locus) [14]. This MdMYB10 allele contains a minisatellite-like structure comprising six tandem repeats located in the promoter region (R6-MdMYB10), while in white-flesh cultivars, only one repeat occurs (R1-MdMYB10). The R6 repeat sequences are self-binding sites of the MdMYB10 protein and are positively correlated with the self-activating activity of its promoter causing anthocyanin ectopic accumulation [15]. In this respect, R6-MdMYB10 is a prerequisite for type 1 red-flesh development. MdMYB110a, a MdMYB10 paralog that arose from a whole-genome duplication event, is responsible for type 2 red-flesh development [13].

Numerous studies have identified additional MYB TFs that positively or negatively regulate anthocyanin synthesis in apples [11]. Other TFs, such as MdbHLH3, MdbHLH33, and WD40 proteins, are involved in anthocyanin synthesis through the formation of the ternary MBW complex [11]. A QTL analysis identified a region associated with red-flesh on linkage group (LG) 16 in a F1 biparental population [16], which colocalized with MdLAR1, a key enzyme in the flavonoid biosynthesis pathway [17]. Transcriptomic analyses revealed differentially expressed genes associated with the flavonoid pathway between red-flesh and white-flesh apples [18], as well as between different sectors of the same bicolor fruit [19]. The WRKY-family TF MdWRKY11 has also been identified as determinant in flavonoid and anthocyanin synthesis [20]. Furthermore, an extreme-phenotype genome-wide association study (XP-GWAS) identified several genetic regions involved in red-flesh color in apples [21].

In fruit species, transposable elements (TEs) have been found in the promoter region of MYB factors, leading to significant modifications in gene expression with consequences on anthocyanin ectopic accumulation [22, 23]. Another activation–repression system has been characterized in fruit species from the *Actinidia* genus and involves an interplay between positive contributions of a MYB TF and negative regulation by miRNA in anthocyanin synthesis and distribution [24].

In red-flesh apple, despite the presence of R6-MdMYB10, there is wide variation in the intensity and distribution of red-flesh pigmentation [25, 26]. R6-MdMYB10 is needed for red-flesh development but other genetic factors modulate red-flesh color expression and lead to important phenotypic variation. While previous studies focused on total anthocyanin contents as a phenotypic trait reflecting flesh color, it is essential to adopt a more quantitative approach to better understand the genetic mechanisms of anthocyanin synthesis in apples. Some genetic studies used rough color intensity visual notation as a phenotypic trait; however, red-flesh pigmentation in apples is complex, leading to continuous variation within and among genotypes [25]. The use of quantitative color descriptors, rather than qualitative indices, is likely to assist in detecting new QTLs [27] and understanding the interactions among these loci.

In this study, we conducted a pedigree-based QTL analysis (PBA-QTL) on five F1 red-flesh apple progenies (452 genotypes), supplemented with genetic data from parents, grandparents, and founders (totaling 544 genotypes). We used quantitative color descriptors (a^*^ parameter for red color intensity) and relevant biochemical factors (anthocyanin, flavan-3-ol and flavonol contents) as phenotypic traits to unravel the genetic architecture of red-flesh color in apples. This study aims to provide new insights into the genetic determinism of red-flesh pigmentation in apples and complete the current genetic model.

## Results

### Phenolic, color variability, and relationship between traits

**Figure 1:**
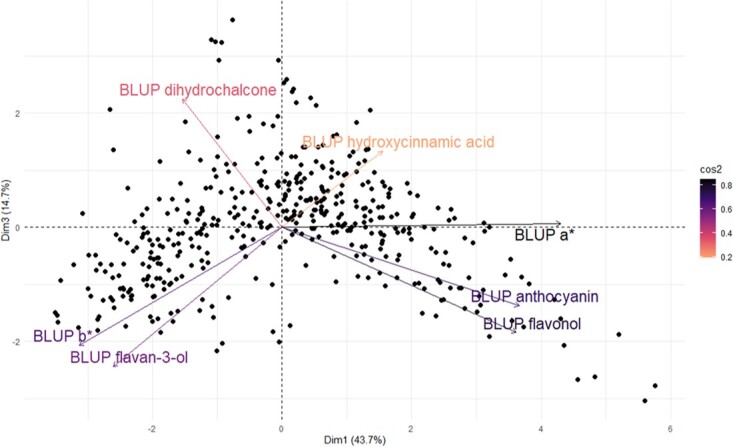
Principal components analysis of color descriptors and phenolic compounds for 432 genotypes from the five F1 hybrid families. The biplot shows the first and third PCA components. PCA loadings of the explanatory variables are colored according to their cos^2^ values (squared cosines represent the correlation of the variables with each principal component), and genotype scores are colored in black (one dot represents one genotype).

Hybrid phenotypes were distributed along a continuous gradient from white-off to dark-red flesh as observed in [25]. a^*^ values varied from −8.1 to 51.8 (Table S1) and exhibited broad-sense heritability over years (*h*^2^ = 0.86). b^*^ values were also repeatable from 2022 and 2023 with *h*^2^ = 0.685 (values oscillating from 1.3 to 22.2 among samples). Anthocyanin, flavan-3-ol, and flavonol contents varied among hybrid samples from 2.96 to 2148.6, 16.09 to 3677.76, and 0.47 to 34.7 μg per gram of dry weight, respectively (considering 2021 and 2022 raw data) (Table S5). Phenolic contents and color descriptors demonstrated robust broad-sense heritability (0.53 $<$*h*^2^$<$0.86) (Table 1). The association between phenolic profile and color expression was confirmed through PCA (Fig. 1) and correlation analysis (Fig. S1). Preliminary results in RF1–1 hybrid progeny identified biochemical factors (anthocyanins, flavan-3-ols, flavonols, and pH) involved in color expression [25]. In this study, we confirm their correlations in the five families.

**Table 1:** Broad-sense heritability of color descriptors estimated in 2021, 2022 and 2023 and phenolic compounds quantified in 2021 and 2022.

Phenotypic trait	Broad-sense heritability
a^*^	0.86
b^*^	0.69
Anthocyanin	0.70
Flavan-3-ol	0.83
Flavonol	0.63
Hydroxycinnamic acid	0.76
Dihydrochalcone	0.53

PCA confirmed between-trait relationships (Fig. 1). First and third axis accounted for 43.7% and 14.7% of the total variance, respectively. Second dimension (17.4% of the total variance) where mostly associated with BLUP dihydrochalcone and BLUP hydroxicinnamic acid variations and showed no clear correlations with BLUP a^*^ and BLUP b^*^ (Fig. S2). Genotypes with high BLUP a^*^ displayed elevated anthocyanin and lower flavan-3-ol contents compared to those with low BLUP a^*^, as illustrated in Fig. 1. As expected, anthocyanin content showed a high correlation with BLUP a^*^ (0.731 - Fig. S1). Flavonol and flavan-3-ol also exhibited correlations with BLUP a^*^ (0.638 and − 0.461, respectively). Flavonol content displayed a positive correlation with anthocyanin content (Pearson correlation = 0.66). *x*–*y* plot between BLUP anthocyanin and BLUP flavan-3-ol confirmed these two trends between high anthoycanin/low flavan-3-ol profiles and, inversely, low anthocyanin/high flavan-3-ol profiles (Fig. S1). Hydroxycinnamic acids and dihydrochalcones were weakly associated with BLUP a^*^ (0.23 and − 0.23) and, consequently, were not included in the proposed genetic model associated with red-flesh color development.

### QTL detection

**Figure 2:**
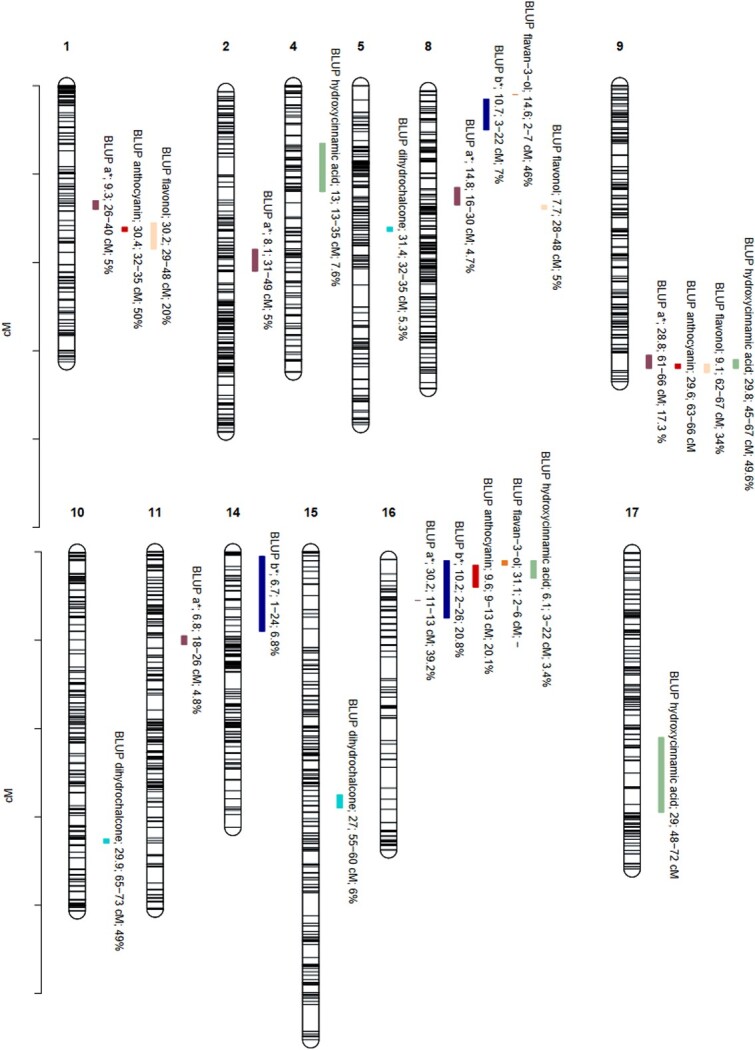
Positions of QTLs controlling flesh color parameters and phenolic compounds. Number of linkage group (LG) are indicated above each group. Positions of SNPs are indicated on the genetic map. Name of the trait is following by Bayes Factor (BF); QTL region and phenotypic variance explained (PVE). Missing PVE indicated inability of QTL model to estimate an accurate value.

QTLs were detected for all phenolic and color traits (Fig. 2. Tables 2 and S2). A total of 24 BLUP-associated QTLs were detected with strong evidence (Bayes Factor; BF; 2lnBf$_{10}>5$) for the seven traits. For BLUP a^*^, BLUP anthocyanin, and BLUP flavan-3-ol, two QTLs were detected at the top of LG16, suggesting a 3-alleles model given that the two putative QTL regions were very close together ($<5$ cM) as discussed in [28, 29].

Two regions each contained clusters of several QTLs. At the bottom of LG9, QTLs were detected for BLUP a^*^, BLUP anthocyanin, BLUP flavanol, and BLUP hydroxycinnamic acid. At the top of LG16, QTLs were detected for BLUP a^*^, BLUP anthocyanin, BLUP flavan-3-ol, and BLUP hydroxycinnamic acid (Fig. 2; Table 2).

Six QTLs were detected for BLUP a^*^, across six linkage groups. The proportion of phenotypic variance (PVE) explained by these ranged from 4.8% to 39.2% (Table 2). These six regions were then investigated for candidate genes (Table 3). An additional QTL, on LG7 (PVE = 3.3%) was detected in 2022 only (data not shown). Four QTLs were detected for BLUP hydroxycinnamic acid, three QTLs each for BLUP b^*^, BLUP anthocyanin, BLUP flavonols and BLUP dihydrochalcone, and two QTLs detected for BLUP flavan-3-ols (Fig. 2; Table 2).

**Table 2:** Summary of BLUP color and phenolic QTLs.

Trait^a^ (BLUP)	LG^b^	BF (1 vs. 0)^c^	BF (2 vs. 1)^d^	QTL interval (cM)^e^	Mode (cM)^f^	PVE (%)^g^
BLUP a^*^	1	9.3	−2.6	[26,40]	29	5
	2	8.1	−0.6	[31,49]	46	5
	8	14.8	−0.7	[16,30]	28	4.7
	9	28.8	4.2	[61,66]	65	17.3
	11	6.8	−3.7	[18,26]	21	4.8
	16	-	30.2	[11,13]	12	39.2
BLUP b^*^	8	10.7	−0.7	[3,22]	11	7
	14	6.7	0.5	[1,24]	23	6.8
	16	-	10.2	[2,26]	16	20.8
BLUP anthocyanin	1	30.4	−4.5	[32,35]	34	50
	9	29.6	1.9	[63,66]	65	-
	16	-	9.6	[9,13]	12	20.1
BLUP flavan-3-ol	8	14.6	−0.7	[2,7]	3	46
	16	-	31.1	[2,6]	4	-
BLUP flavonol	1	30.2	−1	[29,48]	38	20
	8	7.7	0.6	[28,48]	29	5
	9	-	9.1	[62,67]	67	34
BLUP hydroxycinnamic acid	4	13	2	[13,35]	27	7.6
	9	29.8	1.1	[46,67]	66	49.6
	16	6.1	0.5	[3,22]	7	3.4
	17	29	3	[48,72]	60	-
BLUP dihydrochalcone	5	-	31.4	[32,35]	34	5.3
	10	29.9	−9.6	[65,73]	66	49
	15	27	4.5	[55,60]	58	6

a BLUP calculated for the phenotypic trait.

b Linkage group.

c Chromosome-wide Bayes factor (2lnBF10) for a 1 QTL vs. 0 QTL model, with Bayes Factor (BF) $>$ 2, 5, and 10 indicating positive, strong and decisive evidence, respectively, for the presence of one QTL.

d Chromosome-wide Bayes factor (2lnBF10) for a 2 QTLs vs. 1 QTL model, with BF $>$ 2, 5, and 10 indicating positive, strong and decisive evidence, respectively, for the presence of one QTL.

e QTL interval defined as consecutive 2 cM bins (chromosomal segments used by and reported from FlexQTL™) with BF $>$ 5.

f Mode of QTL interval, representing the most probable QTL position.

g Estimated proportion of phenotypic variance explained (PVE) by QTL.

### Haplotype analysis

Haplotype analysis was conducted on LG9-QTL and LG16-QTL. The LG9-QTL haploblock (H9) was constructed from 2 SNPs at 64 cM and colocalized with MdMYB10 (Table S3). Three haplotypes were identified at this QTL locus and denoted as R1, R1’, and R6. Each hybrid had at least one R6 haploallele, coinciding with the presence of R6-MYB10. This is due to selection for red-leaf color at the seedling stage. In the RF1–5 family, which was the sole R1R6 × R1R6 cross, R6-homozygotes were detected. R6R6 diplotypes exhibited significantly higher red-flesh intensity (*P*$<$ 0.05; Fig. 4) with BLUP a^*^ mean values of 51.79 against 32.92 for R1R6 diplotypes.

**Figure 3:**
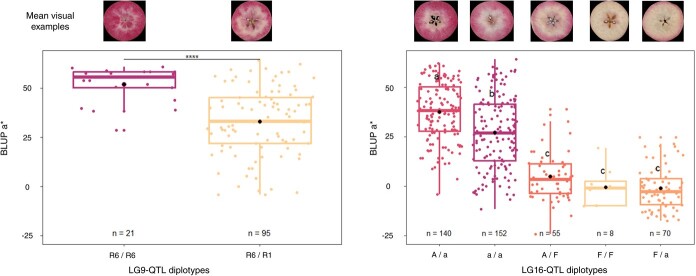
Distribution of BLUP a^*^ for RF1–5 offspring haplotypes for LG9-QTL (left) and the five F1 offspring haplotypes for LG16-QTL (right). The median (denoted by a horizontal bar in the box), the 25th percentile (denoted by the bottom edge of the box), the 75th percentile (denoted by the top edge of the box), the mean (denoted by a black dot), and the dots indicate single observations. Number of genotypes (n) are listed below. Level of significance based on two sample *t*-test is indicated for LG9-QTL haplotypes (^****^ = *P*$<$ 0.0001). Significantly different phenotypic means between segregating classes are identified by different letters (Tukey HSD, *P*$<$ 0.05). Visual examples of group phenotypic means are shown above.

**Figure 4:**
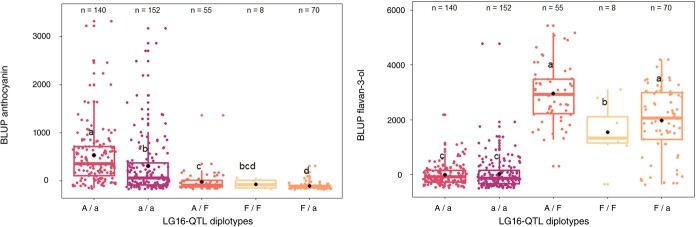
Distribution of BLUP anthocyanin and BLUP flavan-3-ol values for F1 offspring haplotypes for LG16-QTL. The median (denoted by a horizontal bar in the box), the 25th percentile (denoted by the bottom edge of the box), the 75th percentile (denoted by the top edge of the box), the mean (denoted by black dot) and the dots indicate single observations. Number of genotypes (n) are listed above. Significantly different phenotypic means between segregating classes are identified by different letters (non-parametric Dwass-Steel test *P*$<$ 0.05).

**Figure 5:**
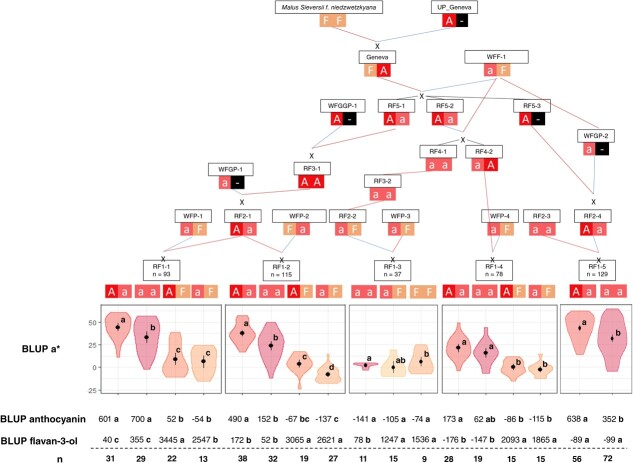
Haplotypes at LG16-QTL for BLUP a^*^, BLUP anthocyanin, and BLUP flavan-3-ol in parental and ancestor cultivars, and in each population. Mean phenotype values of both BLUP of each segregating class detected in each population are shown. Significantly different phenotypic means between segregating classes are identified by different letters (tukey HSD for BLUP a^*^ and non-parametric Dwass–Steel test for BLUP anthocyanin and BLUP flavan-3-ol, *P*$<$ 0.05). ‘n’ refers to the number of genotypes in each diplotype groups. ‘-’ indicated missing data.

**Figure 6:**
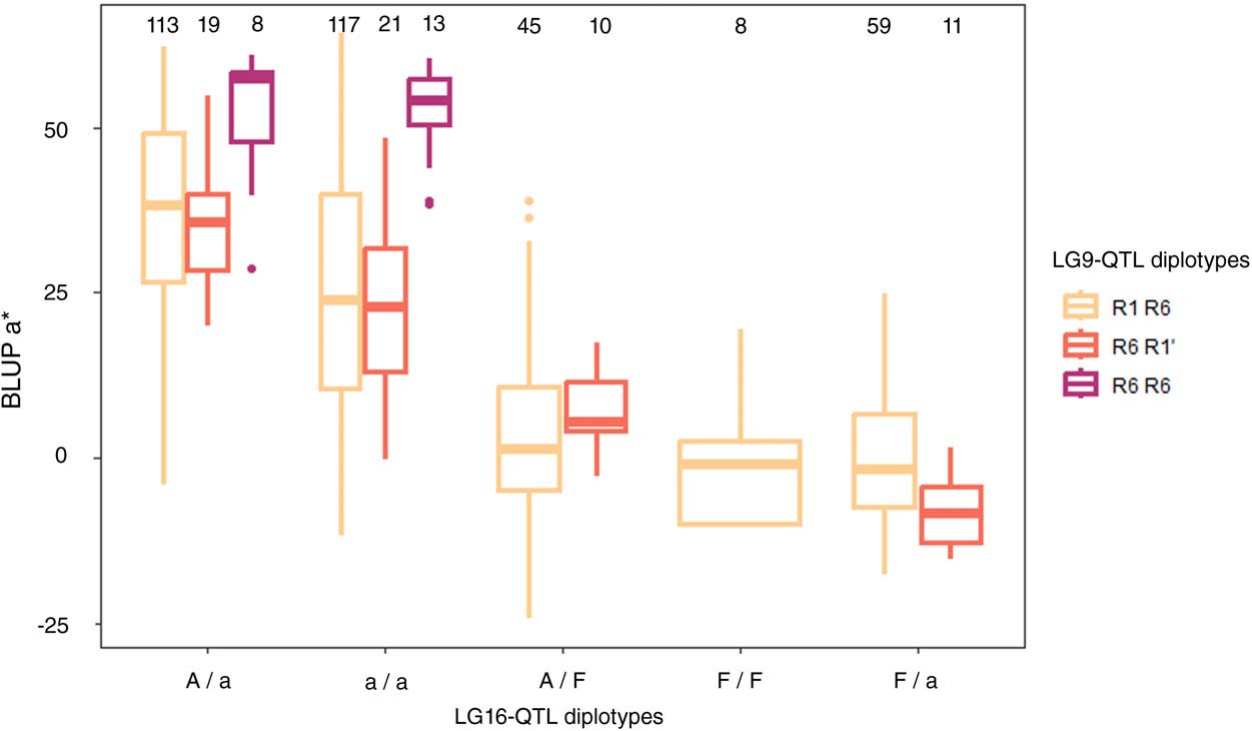
Distribution of BLUP a^*^ for offspring haplotypes for LG9 and LG16 QTLs. The median (denoted by a horizontal bar in the box), the 25th percentile (denoted by the bottom edge of the box), the 75th percentile (denoted by the top edge of the box). Number of genotypes are listed above.

LG16-QTL haploblock (H16) was constructed from 3 SNPs located at 8 cM (Table S3). A 4 cM shift was observed between the QTL mode (12 cM) and the actual QTL position, which can be attributed to the diallelic model implemented by FlexQTL™, while a 3-allele model is generally characterized by two very close putative QTLs. Indeed, three LG16-QTL haplotypes were successfully identified in the hybrid population and designated as H16-A, H16-a, and H16-F for major anthocyanins (A, a) or major flavan-3-ols (F) accumulation, respectively. BLUP a^*^ mean values of diplotype groups A/a, a/a, A/F, F/F, F/a were 37.66, 26.99, 4.95, −0.50 and − 1.20, respectively. Comparison of diplotype effects (Fig. 3) on red-flesh intensity revealed that genotypes without haplotype H16-F had significantly higher BLUP a^*^ than genotypes with. Furthermore, genotypes with H16-A haplotype displayed significantly (*P*$<$ 0.05) higher BLUP a^*^ than those without it. For BLUP a^*^, a dominant effect of H16-F was observed when compared with H16-A and H16-a. Indeed, non-additive genetic effects observed between H16 haplotypes involved dominance of H16-F haplotype at this locus. Using the same H16 haplotypes, a phenotypic comparison of different diplotypes within the hybrid populations was conducted for anthocyanin and flavan-3-ol contents (Fig. 4). BLUP anthocyanin mean values of diplotypes A/a, a/a, A/F, F/F, F/a were 524.3, 300.8, −24.71, −74.08, −106.97, respectively. BLUP flavan-3-ol mean values for these diplotypes were − 13.8, 16.38, 2952.0, 1536.0, and 1984.2, respectively. H16-A and H16-a exhibited a significant (p $<$ 0.05) positive effect on BLUP anthocyanin value. Moreover, genotypes with haplotype H16-A demonstrated higher BLUP anthocyanin values compared to those with only H16-a. In contrast, H16-F displayed a negative effect on BLUP anthocyanin. Conversely, H16-F exhibited a significant (*P*$<$ 0.05) positive effect on BLUP flavan-3-ol. For both BLUP anthocyanin and BLUP flavan-3-ol, a dominant effect of H16-F was observed when compared with H16-A and H16-a. The phenotypic comparison for H16 haplotypes was also carried out within each F1 families and confirmed the genetic model (Fig. 5). Potential additive or epistatic interactions between H9 and H16 were not further investigated, as there were no accumulated diplotypes of H9-R6 homozygote associated with the H16-F haplotype (Fig. 6).

### Source of H16 favorable alleles for red-flesh color

H16 haplotype inheritance was traced back to the different founders of this pedigree. Interestingly, the wild red-flesh founder (*M. sieversii f. niedzwetzkyana*) appeared homozygous for H16-F. The red-flesh cultivar ‘Geneva’ resulted from a cross between *M. sieversii f. niedzwetzkyana* and an unknown white-flesh cultivar. ‘Geneva’ displayed H16-F and H16-A haplotypes, with the favorable H16-A haplotype inherited from this unknown white-flesh parent. The H16-a haplotype was traced back to two white-flesh parent denoted ‘WFF-1’ and ‘WFGP-2’, traced back to a common founder, and considered identical by descent (IBD, Fig. 3).

## Discussion

### Colocalization of b^*^ and phenolic QTLs

LG1-QTL for BLUP flavonol, LG15-QTL for BLUP dihydrochalcone, and LG17-QTL for BLUP hydroxycinnamic acid were colocalized with previously reported QTLs [5, 30, 31]. BLUP b^*^ QTLs on LG8 and LG16 colocalized with Ma and Ma3 loci identified for fruit acidity [29]. Moreover, we found WRKY TFs (MD08G1067700, MD16G1063200, MD16G1066500) in LG8 and LG16 BLUP b^*^ QTL regions (Table S4). We hypothesized that these QTLs could be associated with an anthocyanin hyperchromic shift dependent on vacuolar acidity [25]. Further studies should be conducted to confirm colocalization between b^*^ and pH loci and study the involvement of vacuolar acidity on color expression.

**Table 3:** Putative candidate gene identified within the QTL regions.

Chromosome	QTL region (cM)		Physical position (Mbp)		Gene ID	Annotation
1	26.562	30.517	22.153	23.366	MD01G1117800	ABC transporter, chalcone–flavonone isomerase
2	36.796	48.549	12.763	17.489	MD02G1171300, MD02G1179400	ERF109, UDP-glycosyltransferase
8	21.039	33.82	6.497	12.025	MD08G1121600	FLS
9	60.423	67.009	33.018	37.422	MD09G1278400	MdMYB10
11	18.839	26.88	8.219	11.838	MD11G1100300	Cyanidin 3-O-glucoside 5-O-glucosyltransferase (acyl-glucose)
16	5.012	13.12	2.027	4.15	MD16G1040800, MD16G1040900, MD16G1043500, MD16G1048500	MYB62, bHLH75, ERF118, LAR

### Colocalization of minor a^*^ QTLs and putative candidate genes

Most BLUP a^*^ QTLs were consistent with previously reported QTLs for red-flesh color [16, 21]. LG1 and LG11 BLUP a^*^ QTLs are reported here for the first time, confirming the accuracy and added value of quantitative color analysis for QTL detection. For instance, one QTL, on LG7, was only detected in 2022 at 25–57 cM with a PVE of 3.3%. This QTL region colocalized with MdMYBPA1 which promotes anthocyanin synthesis under low-temperature conditions in red-flesh apples [32]. The existence of a year-related QTL emphasized the influence of environmental conditions on color expression [33].

Minor BLUP a^*^ QTLs were detected on LG1, LG2, LG8, and LG11. Apple genomic data [34] allowed screening for candidate genes associated with these minor QTL regions (Table 3—complete list available in Table S4). A chalcone isomerase (CHI) and a flavonol synthase (FLS), structural genes of the anthocyanin pathway, were found in the QTL interval of LG1-QTL and LG8-QTL, respectively. FLS catalyzes flavonol synthesis, which is consistent with the higher flavonol levels found in red-flesh fruits [35]. Indeed, LG8-QTL colocalized with a BLUP flavonol-related QTL. LG2-QTL colocalized with UDP-glycosyltransferase and ERF109 [36], key regulatory factors of sun-related anthocyanin synthesis in the skin of apple. Indeed, light is one of the most important environmental factors affecting the color of apple fruit and red-flesh color could also be positively regulated by light exposition [11].

All naturally synthesized anthocyanidins are glycosylated on the 3-position by a cytosolic glycosyltransferase to increase stability, solubility, and facilitate transport to the vacuole [10]. LG2-QTL and LG11-QTL colocalized with glycosyltransferase genes, suggesting that red-flesh color acquisition could also be determined by anthocyanin catabolism and not only primary synthesis. Indeed, glycosylation plays a key role in the accumulation of anthocyanins by stabilizing anthocyanins and serving as a signal for transport of anthocyanins to the vacuoles. Furthermore, an ABC transporter gene was found in the LG1-QTL region. ABC transporters are involved in the transfer of glycosylated anthocyanins to the vacuole [10]. Gene expression analysis could enhance our understanding of the complex interplay between anthocyanin synthesis and catabolism occurring during color acquisition throughout fruit development. Wang *et al*. [18, 20] identified key regulatory factors of anthocyanin synthesis by comparing transcriptomes of red-flesh fruits and white-flesh fruits. Extending the comparative transcriptomic analysis of white and red flesh sectors from the same fruit could help us understand the complex interactions between flavonoid synthesis genes and the regulatory factors that orchestrate red-flesh pigmentation in apple.

### Haplotype characterization of LG9 and LG16 QTLs

The LG9 and LG16 QTLs colocalized with previously reported QTLs [14, 16]. LG9-QTL region contains MdMYB10, which R6-MdMYB10 allele is a prerequisite gene for red-flesh color development in apple. LG9-QTL haplotype has been successfully associated with R1-MdMYB10 and R6-MdMYB10. Interestingly, a third haplotype, termed R1’-MdMYB10, was identified in the RF1–2 family. This third allele of the MdMYB10 gene challenges the notion that the MYB10 allelic diversity is constrained to only two alleles. This observation aligns with similar findings related to red-skin color [37]. We did not detect genetic effect of R1’-MdMYB10 with R6-MdMYB10, suggesting that R1 and R1’ have the same genetic effect on red-flesh pigmentation (Fig. S3). Furthermore, this diversity in MdMYB10 alleles is consistent with studies of other MYB10-related anthocyanin synthesis species, such as strawberries [22]. They identified three allelic variants at FaMYB10: FaMYB10.2 activated anthocyanin synthesis resulting in skin and flesh color variations while two independent mutations of FaMYB10 were responsible for white-skin phenotype. Moreover, red-flesh pigmentation was associated with the presence of a transposon insertion in the FaMYB10.2 promoter [22].

Our results indicate that R6-MdMYB10 homozygotes are associated with higher phenotypic values, pointing to an additive effect. The LG16-QTL colocalizes with a leucoanthocyanidin reductaste (LAR) structural gene. LAR catalyzes the conversion of leucocyanidins into flavan-3-ols. The top of LG16 is known to contain a ‘hot-spot’ of QTL for metabolites (mQTLs) associated with Ma locus and many phenolic QTLs [38]. Concomitantly with [38], we detected another QTL for hydroxycinnamic acids despite these metabolites being upstream of LAR substrate. Some MYB and bHLH types TFs have been identified in this region (Table 3). They may be responsible for the QTL for hydroxycinnamic acid.

The presence of the LG16-QTL confirms the competition between anthocyanins/flavan-3-ols as the end product of a common biosynthetic pathway. The H16-F haplotype is associated with a high-flavan-3-ol/low-anthocyanin profile while, the H16-A and H16-a haplotypes are associated with low-flavan-3-ol/high-anthocyanin profile (Fig. S4).

### New insights into genetic mechanisms of red-flesh pigmentation

Our results provide new insights into the genetic mechanisms controling red-flesh development in apple. Indeed, the competition between anthocyanin and flavan-3-ol synthesis is a key factor in red-flesh color acquisition and is mostly driven by the LG16-QTL (PVE = 35.7/39%) in the presence of R6-MdMYB10. The fruits of the wild red-flesh founder (*M. sieversii f. niedzwetzkyana*) are too astringent and sour for a direct use as a dessert apple. Hybridization with white-flesh dessert apples initially aimed to bring the red-flesh locus into elite backgrounds, combining the new phenotype with the desirable organoleptic properties of dessert apples. In this process, favorable alleles for red-flesh color were inherited from the white-flesh background due to negative selection for astringency and bitterness (given by flavan-3-ols), which indirectly favored anthocyanin synthesis. Combination of LG9 and LG16 favorable alleles resulted in a red-flesh ideotype with high coloration and low astringency. However, we only evaluated soluble tannins in our samples. To extend these results, estimation and measurement of polymerized procyanidins by thyolisis [5] should provide a better understanding of the balance between anthocyanin and flavan-3-ol synthesis in red-flesh apple. Further studies could also consider the genotype × environment interactions that would help breeders to select ideotypes suited for most growing conditions.

## Conclusion

In this study, we identified a total of 24 QTLs controling red-flesh color (a^*^ and b^*^ color data) and phenolic profiles (anthocyanins, flavan-3-ols, flavonols, hydroxycinnamic acids, and dihydrochalcone). Thanks to the development of a quantitative approach to characterize fruit flesh color, we detected two previously unidentified QTLs linked to red-flesh color on LG1 and LG11. We confirmed the existence of a major QTL (PVE = 35.7/39.2%) on LG16 controlling red-flesh color in apple [16, 21] and characterized the genetic mechanisms related to anthocyanin/flavan-3-ol balance and red-flesh color enhancement. Homozygotes for R6-MdMYB10 alleles, which colocalized with MdMYB10, reached higher phenotypic values than heterozygotes. However, interactions between LG9 and LG16 QTLs were not studied given that there was no combination of H9-R6 homozygotes with the H16-F haplotype. However, the combination of R6-MdMYB10 homozygosity with favorable LG16-QTL haplotypes (H16-A and H16-a) determined a new ideotype that could be targeted for red-flesh breeding. These results highlight the necessity for breeders to consider white-flesh parents during selection, as positive or negative alleles for red-flesh color can be inherited from the nonred genetic background.

## Materials and methods

### Plant materials

A total of 452 genotypes from five interconnected (common parent and/or grandparent) F_1_ families were used in this study (Fig. S5). Four families resulted from crosses between a type 1 red-flesh and a white-flesh parent (RF1–1 to RF1–4, plantation year: 2018), and one family resulted from a cross between two type 1 red-flesh parents (RF1–5, plantation year: 2017). Genotypes were selected at the seedling stage for red-leaf color (phenotypic marker of MdMYB10 [14]) and apple scab. Consequently, all hybrid genotypes contained at least one R6-MdMYB10 allele. Each genotype is represented by one tree. Fruit harvest was conducted over three years (2021, 2022, and 2023) from August to October in IFO orchard (L’Anguicherie, 49 140 Seiches-sur-le-Loir, France/GCS: 47°37′52.5”N 0°19′38.4”W). For each genotype harvested at maturity (brix values varying from 13 to 22; starch index between 6 and 8) four representative fruits were dedicated to image analysis for estimation of the red-flesh intensity and distribution and four fruits were sampled for the determination of phenolic compounds. Fruits that were positioned in the middle of the tree, at similar light exposure, same developmental stage, and similar diameter were picked preferentially to limit intra- and inter-tree bias.

### Phenotypic data

Images of transversal sections of four fruits per genotype were acquired using an RGB flatbed scanner Canon LIDE 400. An image analysis pipeline was used to estimate color descriptors from RGB images [25]. Fruit sampling, metabolite extraction, detection, and quantification were performed by UPLC-UV as indicated in [25]. Ten phenolic compounds were quantified using calibration with authentic standards and contents were expressed in μg per gram of dry weight. Then, concentrations per polyphenol classes were calculated as follows: hydroxycinnamic acids (sum of chlorogenic acid and 4-*p*-coumaroylquinic acid contents), flavan-3-ol monomers and oligomers/proanthocyanidins (catechin, epicatechin, procyanidin B1, procyanidin B2, procyanidin C1), anthocyanins (cyanidin 3-galactoside – the most abundant anthocyanin in red-flesh apple, with a proportion of over 80%), flavonols (quercetin 3-galactoside), and dihydrochalcones (phloridzin).

### Statistical analysis

Best linear unbiased prediction (BLUP) was estimated for each trait. BLUP is a standard method for estimating random effects of a mixed linear model [39] of the form:


$$ y= X\beta + Zu+e $$

where *y* is the vector of observations (phenotypic scores), $\beta$ and u are vectors of fixed (year effect) and random effects (genotypic values), respectively, *X* and *Z* are the associated design matrices, and e is a random residual vector. The random effects are estimated by BLUP [39]. Because of our experimental design, not all environmental effects could be considered (tree and genotype effects were confounded), and BLUPs of across-year phenotypic values per genotype approximated the true genotypic values.

For a^*^ parameter, BLUPs were estimated from 2021–2022–2023 data. For b^*^ parameter and each phenolic class, BLUPs were calculated from 2021 and 2022 data. Each BLUP was denoted by ‘BLUP trait name’ and used for QTL detection.

Broad-sense heritability of each measured trait was estimated by intra-class analysis [40] with the following formula: *h*^2^ = ${\sigma}_g^2$/(${\sigma}_g^2$+${\sigma}_r^2$) where ${\sigma}_g^2$ and ${\sigma}_r^2$ were the individual genetic and residual variances, respectively.

Principal component analyses (PCA) were carried out to investigate the relation between BLUP for colors descriptors and BLUP for phenolic profiles with the R package ‘FactoMineR’. Pairwise correlation plot for BLUP values was generated with ‘GGally’ package, a ggplot2 extension (Fig. S1). The phenolic compounds correlated with flesh color variation were then considered for further genetic analysis.

### Genotypic data

Hybrids and their parents and ancestors were genotyped using the Illumina Infinium 20 K SNP array for Apple [41]. The raw SNP data was initially processed into the GenomeStudio software v2.0 and SNP data curation was performed as described by Vanderzande *et al*. [42]. After filtering null alleles and alleles with reduced binding affinity across our populations with ASSIsT [43], 8827 SNP markers were considered for further analysis. Their physical position on the Malus genome [34] was taken from integrated genetic linkage (iGL) map for Illumina Infinium 20 K SNP array [44]. A total of 4220 informative SNP were finally retained. SNP markers were well distributed over the 17 chromosomes. Founders were added in our pedigree from public genotyping data on GDR [45]. Thus, we were able to trace marker segregation from our genotypes to the wild red-flesh accession *M. sieversii f. niedzwetzkyana*.

### Haplotype determination

The R package ‘PediHaplotyper’ v.1.0 was used to construct haplotypes [46], resulting in 1104 haploblocks. Maximum size of each haploblock was limited to 1 cM. Identical haplotypes were described as IBD if they could be traced back to a common ancestor, whereas identical haplotypes that could not be traced back to a common ancestor were considered identical-by-state (IBS). Missing SNP data was imputed whenever possible by examining both progenies and ancestors [47]. Haplotypes that could not be resolved were excluded from further analysis.

### QTL detection

QTL analyses were performed using the FlexQTL™ software, which implements PBA-QTL analysis via Markov Chain Monte Carlo (MCMC) simulation (www.flexqtl.nl) [48]. Parameters for the analyses are reported in Supplementary Table S5. Analyses reached adequate MCMC convergence. Two replicate runs with different starting seed numbers were conducted to ensure reproducibility of QTL detection [47, 49]. The significance and stability of a putative QTL were assessed using Bayes Factor (BF; 2lnBF10) and posterior intensity values. Evidence for a QTL was categorized as positive, strong, or decisive based on BF ranges: 2–5, 5–10, and above 10, respectively [49]. QTL intervals were defined as consecutive 2-cM chromosome segments (bins) that had a BF above 5 and the mode within a given QTL region was considered the most probable QTL position [49]. The proportion of PVE explained by a QTL was estimated by dividing the reported variance explained by the whole PVE [29]. Colocalization between phenolic QTL and color QTL were then targeted to unravel genetic mechanisms of red-flesh color development.

Positive/negative alleles were defined via analyses of SNP haplotypes within QTL intervals. Comparison of diplotypes (diploid combinations of two haplotypes) were used to infer haplotype effects. To determine if mean a^*^ BLUP was significantly different for presence versus absence of a given QTL haplotype, one-way analysis of variance (ANOVA) and Tukey’s honest significant difference (HSD) were calculated. To determine whether BLUPs associated with phenolic profile (anthocyanin and flavan-3-ol content) were significantly different between the functional diplotypes, nonparametric Dwass-Steel tests were carried out for each compound [47]. Nonparametric Dwass-Steel test were also applied for phenotypic comparison between QTL haplotypes within each F1 family to confirm model consistency. Tidyverse R collection was used to perform data management and generate plots. For graphical display, colorblindness-friendly colors were provided by ‘viridis’ package. Statistical analysis were performed combining R package ‘stats’, ‘rstatix’, and ‘biostats‘.

### Candidate gene search in the apple genome

To investigate candidate gene colocalization in BLUP a^*^ QTL regions (Table S4), genes and TFs involved in anthocyanin synthesis were searched among QTL region (Table 3) using the ‘GGDH13.v1.1’ apple genome [34] as reference. Genetic positions were defined as the boundaries of the two inner haploblocks surrounding the QTL region.

## Supplementary Material

Web_Material_uhae171

## Data Availability

The data underlying this article cannot be shared publicly due to privacy of pedigree and genotyping data. The data will be shared on reasonable request to the corresponding author.

## References

[1] Migicovsky Z , Gardner KM, Richards C. et al. Genomic consequences of apple improvement. Hortic Res. 2021;8:9 33384408 10.1038/s41438-020-00441-7 PMC7775473

[2] Wang N , Jiang S, Zhang Z. et al. Malus sieversii: the origin, flavonoid synthesis mechanism, and breeding of red-skinned and red-fleshed apples. Hortic Res. 2018a;5:70 30345062 10.1038/s41438-018-0084-4 PMC6186759

[3] Allan AC , Hellens RP, Laing WA. Myb transcription factors that colour our fruit. Trends Plant Sci. 2008;13:99–102 18280199 10.1016/j.tplants.2007.11.012

[4] Zhang Y , Butelli E, Martin C. Engineering anthocyanin biosynthesis in plants. Curr Opin Plant Biol. 2014;19:81–90 24907528 10.1016/j.pbi.2014.05.011

[5] Verdu CF , Guyot S, Childebrand N. et al. Qtl analysis and candidate gene mapping for the polyphenol content in cider apple. PLoS One. 2014;9:e107103 25271925 10.1371/journal.pone.0107103 PMC4182701

[6] Lin Q , Chen J, Liu X. et al. A metabolic perspective of selection for fruit quality related to apple domestication and improvement. Genome Biol. 2023;24:95 37101232 10.1186/s13059-023-02945-6 PMC10131461

[7] Farneti B , Masuero D, Costa F. et al. Is there room for improving the nutraceutical composition of apple? J Agric Food Chem. 2015;63:2750–9 25723891 10.1021/acs.jafc.5b00291

[8] Bars-Cortina D , Macià A, Iglesias I. et al. Phytochemical profiles of new red-fleshed apple varieties compared with traditional and new white-fleshed varieties. J Agric Food Chem. 2017;65:1684–96 28191939 10.1021/acs.jafc.6b02931

[9] Ceci AT , Bassi M, Guerra W. et al. Metabolomic characterization of commercial, old, and red-fleshed apple varieties. Metabolites. 2021;11:378 34208114 10.3390/metabo11060378 PMC8230845

[10] Buhrman K , Aravena-Calvo J, Ross Zaulich C. et al. Anthocyanic vacuolar inclusions: from biosynthesis to storage and possible applications. Front Chem. 2022;10:913324 35836677 10.3389/fchem.2022.913324 PMC9273883

[11] Chen Z , Yu L, Liu W. et al. Research progress of fruit color development in apple (malus domestica borkh.). Plant Physiol Biochem. 2021;162:267–79 33711720 10.1016/j.plaphy.2021.02.033

[12] Lloyd A , Brockman A, Aguirre L. et al. Advances in the myb-bhlh-wd repeat (mbw) pigment regulatory model: addition of a wrky factor and co-option of an anthocyanin myb for betalain regulation. Plant Cell Physiol. 2017;58:1431–41 28575507 10.1093/pcp/pcx075 PMC5914458

[13] Chagné D , Lin-Wang K, Espley RV. et al. An ancient duplication of apple myb transcription factors is responsible for novel red fruit-flesh phenotypes. Plant Physiol. 2012b;161:225–39 23096157 10.1104/pp.112.206771 PMC3532254

[14] Chagné D , Carlisle CM, Blond C. et al. Mapping a candidate gene (mdmyb10) for red flesh and foliage colour in apple. BMC Genomics. 2007;8:212 17608951 10.1186/1471-2164-8-212 PMC1939713

[15] Espley RV , Brendolise C, Chagne D. et al. Multiple repeats of a promoter segment causes transcription factor autoregulation in red apples. Plant Cell. 2009;21:168–83 19151225 10.1105/tpc.108.059329 PMC2648084

[16] Yang C , Sha G, Wei T. et al. Linkage map and qtl mapping of red flesh locus in apple using a r1r1×r6r6 population. Hortic Plant J. 2021;7:393–400

[17] Kumar S , Garrick DJ, Bink MC. et al. Novel genomic approaches unravel genetic architecture of complex traits in apple. BMC Genomics. 2013;14:393 23758946 10.1186/1471-2164-14-393 PMC3686700

[18] Wang N , Zheng Y, Duan N. et al. Comparative transcriptomes analysis of red- and white-fleshed apples in an f1 population of malus sieversii f. niedzwetzkyana crossed with m. domestica ‘Fuji’. PLoS One. 2015;10:e0133468 26207813 10.1371/journal.pone.0133468 PMC4514764

[19] Li H , Duan S, Sun W. et al. Identification, through transcriptome analysis, of transcription factors that regulate anthocyanin biosynthesis in different parts of red-fleshed apple ‘may’ fruit. Hortic Plant J. 2022;8:11–21

[20] Wang N , Liu W, Zhang T. et al. Transcriptomic analysis of red-fleshed apples reveals the novel role of mdwrky11 in flavonoid and anthocyanin biosynthesis. J Agric Food Chem. 2018b;66:7076–86 29909630 10.1021/acs.jafc.8b01273

[21] Kumar S , Deng CH, Molloy C. et al. Extreme-phenotype gwas unravels a complex nexus between apple (malus domestica) red-flesh colour and internal flesh browning. Fruit Res. 2022;2:1–14

[22] Castillejo C , Waurich V, Wagner H. et al. Allelic variation of myb10 is the major force controlling natural variation in skin and flesh color in strawberry (fragaria spp.) fruit. Plant Cell. 2020;32:3723–49 33004617 10.1105/tpc.20.00474 PMC7721342

[23] Tian Y , Thrimawithana A, Ding T. et al. Transposon insertions regulate genome-wide allele-specific expression and underpin flower colour variations in apple (malus spp.). Plant Biotechnol J. 2022;20:1285–97 35258172 10.1111/pbi.13806 PMC9241373

[24] Wang W , Moss SMA, Zeng L. et al. The red flesh of kiwifruit is differentially controlled by specific activation-repression systems. New Phytol. 2022;235:630–45 35348217 10.1111/nph.18122

[25] Bouillon P , Fanciullino A-L, Belin E. et al. Image analysis and polyphenol profiling unveil red-flesh apple phenotype complexity. Plant Methods. 2024;20:71 38755652 10.1186/s13007-024-01196-1 PMC11100172

[26] van Nocker S , Berry G, Najdowski J. et al. Genetic diversity of red-fleshed apples (malus). Euphytica. 2011;185:281–93

[27] Underhill AN , Hirsch CD, Clark MD. Evaluating and mapping grape color using image-based phenotyping. Plant Phenomics. 2020 10.34133/2020/8086309 PMC7706331 33313563

[28] Kostick SA , Teh SL, Norelli JL. et al. Fire blight qtl analysis in a multi-family apple population identifies a reduced-susceptibility allele in ‘honeycrisp’. Horticulture Research. 2021;8:28 33518709 10.1038/s41438-021-00466-6 PMC7847996

[29] Verma S , Evans K, Guan Y. et al. Two large-effect qtls, ma and ma3, determine genetic potential for acidity in apple fruit: breeding insights from a multi-family study. Tree Genet Genomes. 2019;10:1–17

[30] Chagné D , Krieger C, Rassam M. et al. Qtl and candidate gene mapping for polyphenolic composition in apple fruit. BMC Plant Biol. 2012a;12:12 22269060 10.1186/1471-2229-12-12 PMC3285079

[31] McClure KA , Gong Y, Song J. et al. Genome-wide association studies in apple reveal loci of large effect controlling apple polyphenols. Horticulture Research. 2019;6:107 31645962 10.1038/s41438-019-0190-y PMC6804656

[32] Wang N , Qu C, Jiang S. et al. The proanthocyanidin-specific transcription factor mdmybpa1 initiates anthocyanin synthesis under low-temperature conditions in red-fleshed apples. Plant J. 2018c;96:39–55 29978604 10.1111/tpj.14013

[33] Espley RV , Jaakola L. The role of environmental stress in fruit pigmentation. Plant Cell Environ. 2023;46:3663–79 37555620 10.1111/pce.14684

[34] Daccord N , Celton J-M, Linsmith G. et al. High-quality de novo assembly of the apple genome and methylome dynamics of early fruit development. Nat Genet. 2017;49:1099–106 28581499 10.1038/ng.3886

[35] Wang N , Xu H, Jiang S. et al. MYB12 and MYB22 play essential roles in proanthocyanidin and flavonol synthesis in red-fleshed apple (*Malus sieversii* f*. niedzwetzkyana*). Plant J. 2017;90:276–92 28107780 10.1111/tpj.13487

[36] Ma H , Yang T, Li Y. et al. The long noncoding rna mdlnc499 bridges mdwrky1 and mderf109 function to regulate early-stage light-induced anthocyanin accumulation in apple fruit. Plant Cell. 2021;33:3309–30 34270784 10.1093/plcell/koab188 PMC8505877

[37] Zhang L , Hu J, Han X. et al. A high-quality apple genome assembly reveals the association of a retrotransposon and red fruit colour. Nat Commun. 2019;10:1494 30940818 10.1038/s41467-019-09518-x PMC6445120

[38] Khan SA , Chibon P-Y, de Vos RC. et al. Genetic analysis of metabolites in apple fruits indicates an mqtl hotspot for phenolic compounds on linkage group 16. J Exp Bot. 2012;63:2895–908 22330898 10.1093/jxb/err464 PMC3350913

[39] Piepho HP , Möhring J, Melchinger AE. et al. Blup for phenotypic selection in plant breeding and variety testing. Euphytica. 2007;161:209–28

[40] Xu S . Quantitative Genetics. Springer International Publishing; 2022

[41] Bianco L , Cestaro A, Sargent DJ. et al. Development and validation of a 20k single nucleotide polymorphism (snp) whole genome genotyping array for apple (malus × domestica borkh). PLoS One. 2014;9:e110377 25303088 10.1371/journal.pone.0110377 PMC4193858

[42] Vanderzande S , Howard NP, Cai L. et al. High-quality, genome-wide snp genotypic data for pedigreed germplasm of the diploid outbreeding species apple, peach, and sweet cherry through a common workflow. PLoS One. 2019;14:e0210928 31246947 10.1371/journal.pone.0210928 PMC6597046

[43] Di Guardo M , Micheletti D, Bianco L. et al. Assist: an automatic snp scoring tool for in- and outbreeding species. Bioinformatics. 2015;31:3873–4 26249809 10.1093/bioinformatics/btv446 PMC4653386

[44] Howard NP , Troggio M, Durel C-E. et al. Integration of infinium and axiom snp array data in the outcrossing species malus × domestica and causes for seemingly incompatible calls. BMC Genomics. 2021;22:246 33827434 10.1186/s12864-021-07565-7 PMC8028180

[45] Jung S , Lee T, Cheng C-H. et al. 15 years of gdr: new data and functionality in the genome database for rosaceae. Nucleic Acids Res. 2018;47:D1137–45 10.1093/nar/gky1000 PMC6324069 30357347

[46] Voorrips RE , Bink MCAM, Kruisselbrink JW. et al. Pedihaplotyper: software for consistent assignment of marker haplotypes in pedigrees. Mol Breed. 2016;36:119 27547106 10.1007/s11032-016-0539-y PMC4977329

[47] Powell AA , Kostick SA, Howard NP. et al. Elucidation and characterization of qtls for russet formation on apple fruit in ‘honeycrisp’-derived breeding germplasm. Tree Genet Genomes. 2022;19:5

[48] Bink MCAM , Jansen J, Madduri M. et al. Bayesian qtl analyses using pedigreed families of an outcrossing species, with application to fruit firmness in apple. Theor Appl Genet. 2014;127:1073–90 24567047 10.1007/s00122-014-2281-3

[49] Kostick SA , Luby JJ. Apple fruit size qtls on chromosomes 8 and 16 characterized in ‘honeycrisp’-derived germplasm. Agronomy. 2022;12:1279

