# Supplementary figures and images for: Tracing the color: quantitative trait loci analysis reveals new insights into red-flesh pigmentation in apple (Malus domestica)
Source: Hortic Res. 2024 Jun 27;11(8):uhae171. doi: 10.1093/hr/uhae171 (PMC11301320; doi:10.1093/hr/uhae171)

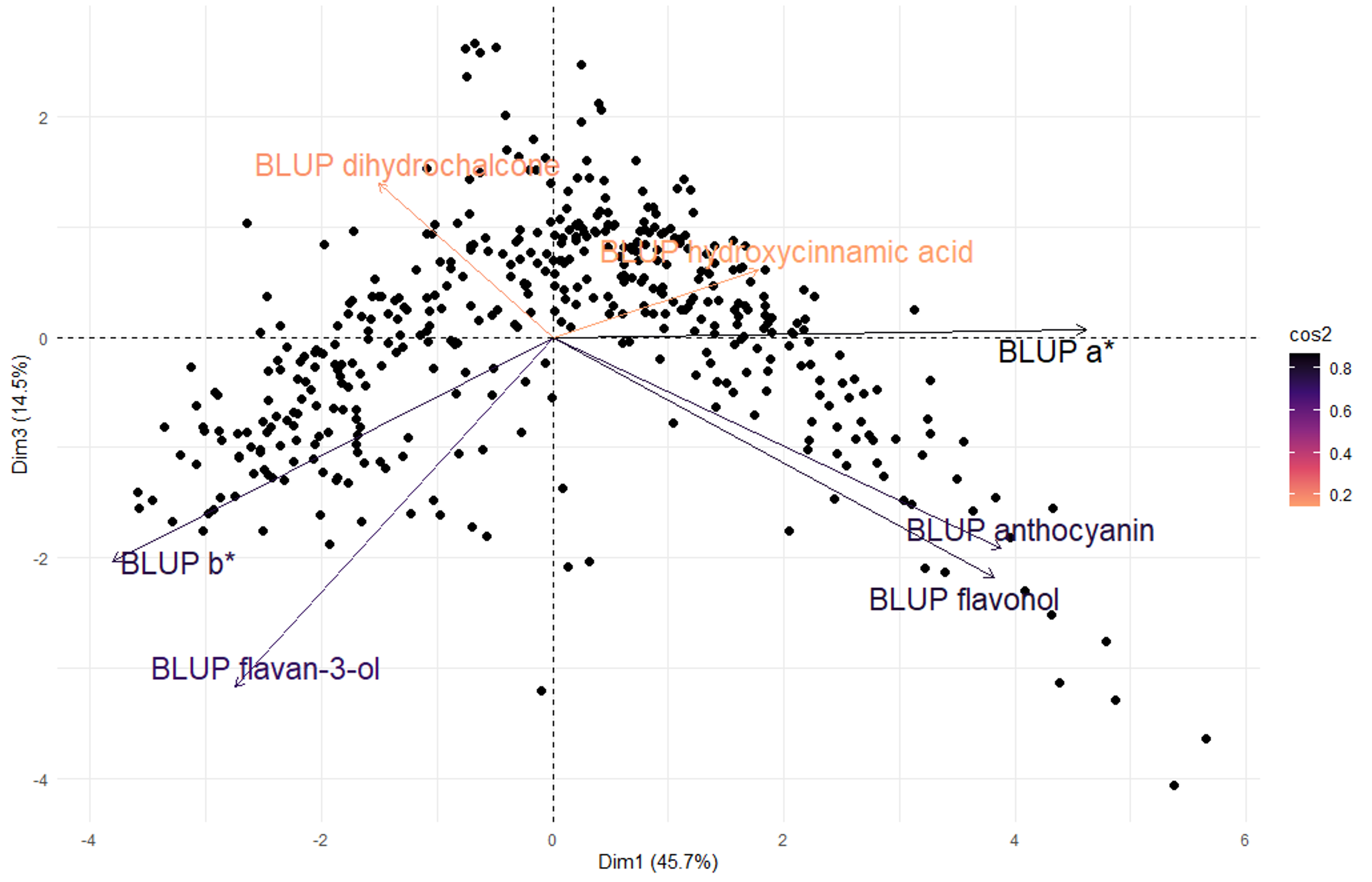

Supplement: Web_Material_uhae171 [file web_material_uhae171.zip › fig1.tif]
